# A Case of Castleman's Disease during the Long-Term Course of Membranous Nephropathy

**DOI:** 10.1155/2023/4926000

**Published:** 2023-02-22

**Authors:** Shuhei Nakajima, Kei Nagai, Akiko Sakata, Joichi Usui, Kunihiro Yamagata, Atsushi Ueda

**Affiliations:** ^1^Department of Nephrology, Hitachi General Hospital, 2-1-1 Jonan-cho, Hitachi, Ibaraki 317-0077, Japan; ^2^Department of Nephrology, Faculty of Medicine, University of Tsukuba, 1-1-1 Tennodai, Tsukuba, Ibaraki 305-8575, Japan; ^3^Department of Pathology, Hitachi General Hospital, 2-1-1 Jonan-cho, Hitachi, Ibaraki 317-0077, Japan

## Abstract

Concomitant with nephrotic syndrome and multicentric castleman's disease (MCD) has only been described in a limited number of small studies and case reports. Among those, none confirmed the renal pathology prior to the onset of MCD, and none of the cases had a history of nephrotic syndrome. A 76 year-old Japanese man visited a nephrologist because of incident nephrotic syndrome. He had previously experienced three episodes of nephrotic syndrome, the last one 13 years ago, and had been diagnosed with membranous nephropathy by renal biopsy. Apart from these previous episodes, he also suffered from systemic lymphadenopathy, anemia, elevated C-reactive protein, polyclonal hypergammopathy, and elevated interleukin (IL)-6. An inguinal lymph node biopsy revealed CD138-positive plasma cells in the interfollicular region. Based on these findings, MCD was diagnosed. Renal biopsy indicated primary membranous nephropathy with spike lesions and bubbling in the basement membranes and deposition of immunoglobulin (Ig) G, IgA, IgM, and phospholipase A2 receptor along the glomerular basement membrane. Corticosteroid monotherapy successfully reduced the edema, proteinuria, and IL-6, but hypoalbuminemia was not sufficiently improved due to castleman's disease and remission of the nephrotic syndrome was not achieved. Later, tocilizumab was administered for remission induction in another facility. To the best of our knowledge, this represents the first report of Castleman's disease with previously diagnosed membranous nephropathy. This case does not provide a causal mechanism for the pathophysiology, but it may be worth suggesting possible involvement of MCD as a trigger for recurrence of membranous nephropathy.

## 1. Introduction

Castleman's disease (CD) is a disorder involving a systemic inflammatory response and multi-organ failure caused by overproduction of interleukin (IL)-6 from proliferated lymph nodes [1]. The clinical symptoms of multicentric CD (MCD) include lymphadenopathy, anemia, hypoalbuminemia, splenomegaly, renal dysfunction, fever [2], and infrequently, renal disease presenting as hematuria, proteinuria, or renal failure.

Renal involvement in MCD has only been described in a limited number of small studies and case reports. A literature review of 64 cases published between 1975 and 2010 revealed amyloidosis as the main pathological characteristic seen on renal biopsy in 39% of cases, followed by membranoproliferative glomerulonephritis in 11%, thrombotic microangiopathy in 8%, and membranous nephropathy (MN) in only 3 cases (5%) [3]. Studies of biopsy-confirmed renal diseases in CD patients from France and China have also shown low frequencies of MN (0/19 cases and 1/11 cases, respectively) [3, 4]. Collectively, nephrotic syndrome concomitant with CD, particularly MN, appears to be an extremely rare condition that may respond to corticosteroid therapy as a monotherapy or in combination with anti-IL-6 antibody treatment and other immunosuppressants to control disease activity [3, 5].

In addition to such rare cases, we recently encountered a patient with CD during the long-term course of previously diagnosed MN.

## 2. Case Presentation

A 76-year-old Japanese man visited a nephrologist with complaints of edema and dyspnea. He had first been diagnosed with nephrotic syndrome at 38 years old. Nephrotic syndrome had recurred at 57 and 63 years old. With the previous recurrence at 63 years old, he underwent renal biopsy and was diagnosed with MN (Ehrenreich-Churg stage II) with granular deposition of IgG, IgA, and IgM along the capillary wall. After the biopsy, he was treated with oral prednisolone (PSL) at 25 mg/day. Ten days later, the PSL dose was increased to 40 mg/day because urinary protein levels remained elevated. Urinary protein gradually decreased, and the patient achieved remission 3 months later. PSL was tapered and discontinued after 2 years of maintenance treatment. Although immunoglobulin (Ig) levels were not available, the ratio of albumin to globulin was 1.29 (total protein, 7.1 g/dL; albumin, 4.0 g/dL), suggesting that no hyperglobulinemia was present after remission at 65 years old.

During follow-up, edema of the lower extremity and pericardial effusion gradually developed, and he was referred to a cardiologist. Laboratory workup then revealed a recurrence of nephrotic syndrome, and he again consulted a nephrologist. Initially, body temperature was normal with no obvious abnormalities in other vital signs. On physical examination, the patient showed pitting edema of the lower extremities and lymphadenopathy bilaterally in the axillary and inguinal regions. Laboratory data showed massive proteinuria (4.4 g/day) and hypoalbuminemia (1.4 g/day) (Table 1), suggesting nephrotic syndrome without hematuria. Notable findings were newly developed polyclonal gammopathies (IgG, 3,468 mg/dL; IgA, 534 mg/dL; and IgM, 284 mg/dL). Anemia and systemic inflammation were indicated from: hemoglobin, 9.8 g/dL; C-reactive protein, 0.63 mg/dL; and IL-6, 22.1 pg/mL (Reference; <7 pg/mL). Computed tomography (CT) showed multiple lymphadenopathies in the mediastinum, inguinal, and axillary regions. Blood culture, tuberculosis tests, tumor markers, and protein electrophoresis of sera and urine, and autoantibodies all yielded negative results.

**Figure 1:**
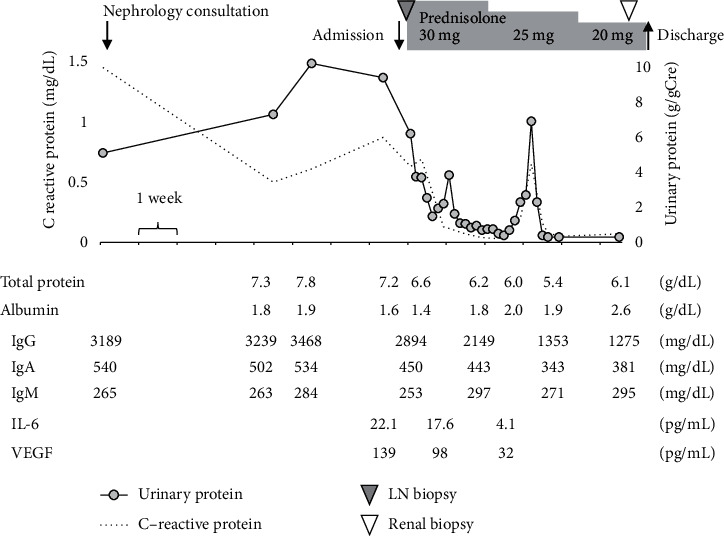
Clinical course of the patient. The patient was referred to a nephrologist with a diagnosis of nephrotic syndrome at 76 years of age. The serum level of immunoglobulins was high in addition to massive urinary protein, and polyclonal gammopathy was identified. Before administration of prednisolone for the treatment of nephrotic syndrome, inguinal lymph node biopsy was performed. Nephrotic syndrome promptly responded to prednisolone and proteinuria improved along with decreased disease markers of Castleman's disease, such as IgG, IgA, C-reactive protein, serum IL-6, and plasma VEGF. During tapering of prednisolone, proteinuria transiently elevated. We performed renal biopsy to evaluate activity of renal involvement for decision making regarding long-term treatment. LN, lymph node; Ig, immunoglobulin; IL-6, interleukin-6; and VEGF, vascular endothelial growth factor.

**Figure 2:**
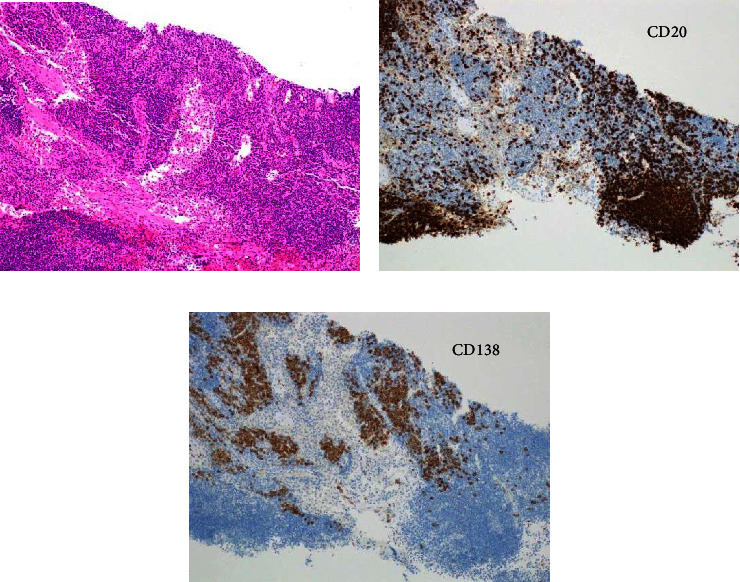
Lymph node pathology. Light microscopic appearance of inguinal lymph node obtained by needle biopsy reveals diffuse interfollicular plasma cell infiltration (hematoxylin and eosin staining (HE)) (a). As hyaline vascular proliferation was not evident, this case present plasma cell type of MCD. Immunohistochemistry reveals CD20-positive lymphocytes in the follicular region (b), while significant CD138-positive plasma cells exist in the interfollicular region (c).

Figure 1 indicates the clinical course of this case. Before administration of PSL to treat nephrotic syndrome, an inguinal lymph node biopsy was performed for a definitive diagnosis. Light microscopy revealed diffuse interfollicular infiltration of plasma cells. Immunohistochemistry revealed CD20-positive lymphocytes in the follicular region and significant CD138-positive plasma cells in the interfollicular region (Figure 2). As hyaline vascular proliferation was not evident, this case presents the plasma cell type of MCD rather than the hyaline-vascular type. Furthermore, additional staining to diagnose malignant lymphoma yielded negative results, and the lack of monoclonality indicated no evidence of deviation in the light chain limitation. Based on polyclonal gammopathy, the pathology of lymphadenopathy, and elevated IL-6, we diagnosed MCD with renal involvement.

**Figure 3:**
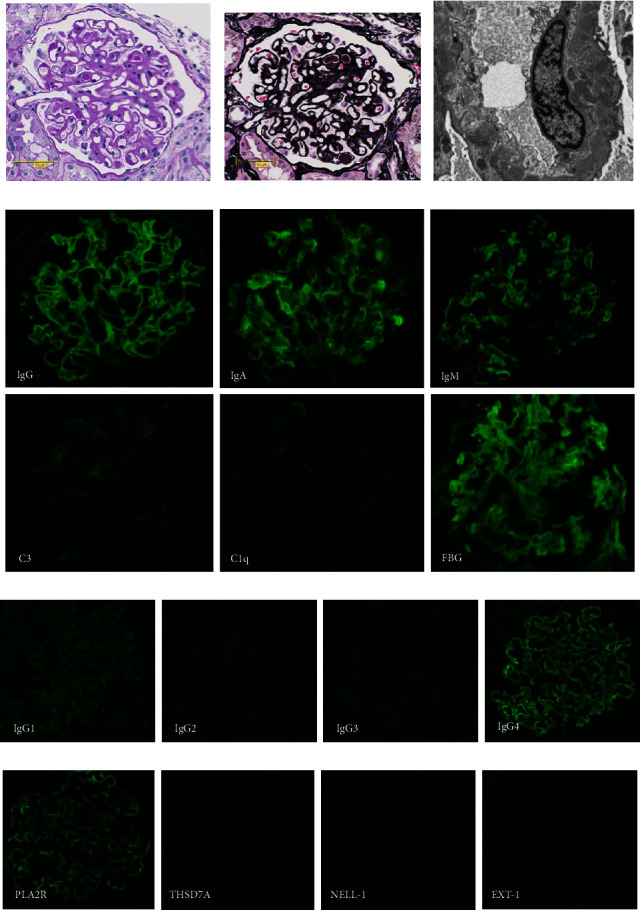
Renal biopsy findings. Periodic acid schiff (PAS) staining shows no obvious mesangial proliferation, crescent, or focal adhesions (a). Periodic acid methenamine silver (PAM) staining shows spikes and bubbling in the basement membranes (b). Electron microscopy reveals subepithelial and intramembrane deposits of homogenous sizes and stages and mild subendothelial enlargement (c). Immunofluorescent findings show marked granular deposit of IgG, IgA, and IgM, but no staining for complement (d). We examined depositions of IgG subclasses (e) and several MN-associated antigens as follows: phospholipase A2 receptor (PLA2R), thrombospondin type-1domain-containing 7A (THSD7A), neural epidermal growth factor-like 1 (NELL-1), and exostosin-1 (EXT-1) (f).

Nephrotic syndrome promptly responded to PSL, and proteinuria improved and reached 0.2 g/day by day 14, along with decreases in disease markers of MCD such as IgG, IgA, C-reactive protein, serum IL-6, and plasma vascular endothelial growth factor (VEGF). At the same time, leg edema, and pericardial effusion gradually improved. During the tapering of PSL, proteinuria was transiently elevated, and we performed a renal biopsy to evaluate the activity of renal involvement and the differential diagnosis of primary or secondary MN. The renal specimen contained 12 glomeruli, with global sclerosis in three. No infiltration of inflammatory cells or mesangial cellular proliferation, crescents, or adhesions were noted (Figure 3(a)). Glomerular capillaries were diffusely and globally thickened and periodic acid methenamine silver staining showed spikes and bubbling in basement membranes (Figure 3(b)). Electron microscopy revealed subepithelial and intramembrane deposits of homogenous size estimated as Ehrenreich–Churg stage II late to III, partially with stage IV, and mild subendothelial enlargement (Figure 3(c)). Immunofluorescent investigations showed marked granular deposition of IgG, IgA, and IgM along the glomerular basement membrane, but no staining for complement (Figure 3(d)). We further assessed IgG subclass and revealed positive staining for IgG4, while faintly stained in IgG1 and negatively stained in IgG2 and IgG3 (Figure 3(e)). Regarding antigen-specific antibody staining, deposition of phospholipase A2 receptor (PLA2R) was positive in this case, while any other representative antigen-specific antibodies were not stained (thrombospondin type-1domain-containing 7A, neural epidermal growth factor-like 1 and exostosin-1) (Figure 3(f)). Finally, he was diagnosed with recurrence of primary MN of Ehrenreich–Churg stage II to III coincident with MCD.

**Figure 4:**
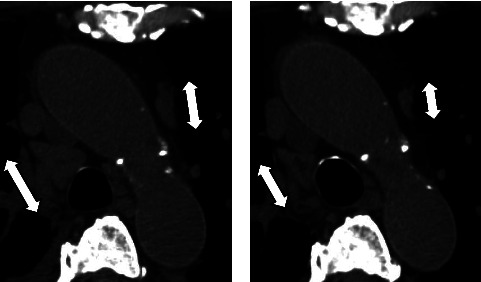
Radiological findings. Computed tomography of the chest before (a) and after treatment (b). Periaortic and lung hilar lymph nodes (two-headed arrow) appear reduced in size.

**Figure 5:**
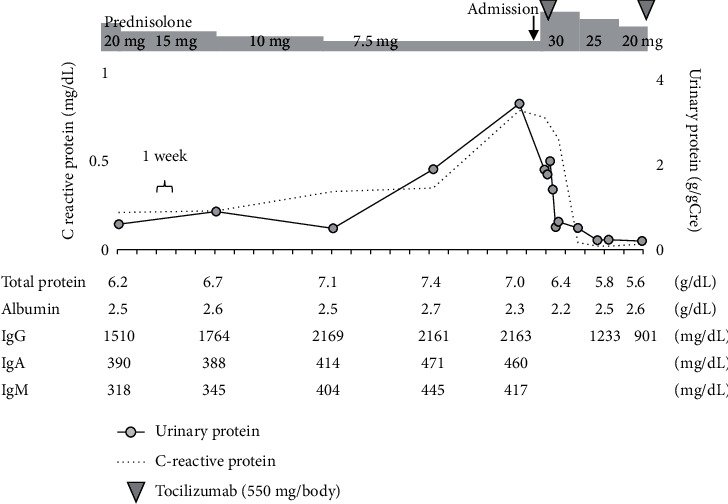
Subsequent clinical course of the patient. In the case, proteinuria was successfully controlled for 6 months with corticosteroid monotherapy after discharge from our hospital. During the process of tapering prednisolone, serum IgG titer began to increase, and polyclonal gammopathy appeared. The patient was referred to another hospital for induction of anti-IL-6 therapy, tocilizumab. The disease markers of MCD such as serum IL-6 and plasma vascular endothelial growth factor were not examined.

At discharge after 6 weeks of hospitalization, we performed a CT to confirm the effect of the lymph node reduction in size throughout the body. The periaortic and lung hilar lymph nodes were reduced in size (Figure 4), as were the lymph nodes in the bilateral axillary and inguinal regions. This case of proteinuria was successfully controlled for 6 months with corticosteroid monotherapy, and proteinuria was kept below 0.5 g/day. However, during the process of tapering PSL to 15 mg/day, the serum IgG titer (bottom; 1,275 mg/dL) began to increase, and at a PSL dose of 7.5 mg/day, urinary protein increased from 0.5 g to 1.9 g, CRP changed from negative to positive (0.33 mg/dL), and polyclonal gammopathy (IgG 2,161 mg/dL, IgA 471 mg/dL, and IgM 445 mg/dL) appeared. The patient was referred to another hospital for induction of anti-IL-6 therapy (Figure 5). Combination therapy with PSL and tocilizumab has successfully decreased concentration of IgG, C-reactive protein, and proteinuria, again.

## 3. Discussion

This case displayed newly developed CD with nephrotic syndrome after remission of previously diagnosed MN. The first nephrotic episode occurred 38 years earlier, with the first recurrence after 19 years, the second recurrence after another 6 years, and renal biopsy only performed at that time. Although IL-6 levels were not measured previously, the level of CRP was negative (<0.1 mg/dL), and there was no evidence of hypergammopathy in the previous episodes. We therefore speculated that differences existed between the third recurrence of nephrotic syndrome (case presentation at 76 years old) and the course of the previous MN episodes (up to 63 years old). Since corticosteroids are expected to suppress lymph node proliferation and immunoglobulin production itself, the remission induction phase in this case was intended to confirm whether corticosteroid monotherapy improved nephrotic syndrome along with decreasing levels of IL-6 and CRP. Unfortunately, hypoalbuminemia was not sufficiently improved, and remission of the nephrotic syndrome was not achieved, but a prompt decrease in urinary protein was achieved.

We reviewed the 11 reported cases of MN with CD published between 1979 and 2021 [3, 5–14]. In some cases, the immunoglobulin deposition patterns in the glomerular capillary wall have not been fully described [3, 5, 9], but the remaining cases demonstrated IgG deposition. In the cases with IgA and IgM staining in renal specimens, they are deposited faintly [6, 7, 14] or not deposited [11, 13]. Therefore, it seems to be rare for IgG, IgA, and IgM to all be deposited as in the present case. Subclasses pattern of IgG in the previous cases are mostly characterized as IgG1 (+), IgG2 (+), IgG3 (−), and IgG4 (−) [8, 10, 11, 13]. In the present case, only IgG4 is stained, suggesting at least the past nephrotic syndrome was primary MN and the current episode can be considered a recurrence of primary MN. In previous cases, the deposition pattern of PLA2R in MN as a result of renal involvement of CD was controversial, but negative [13] or weakly positive [10, 14]. Therefore, examinations for specific antigens of MN in our case revealed predominant PLA2R deposition in the glomeruli, which supports the diagnosis of primary MN.

Among the 11 reported cases of MN, no renal pathology was confirmed prior to the onset of CD. Similarly, none of the cases had any prior history of nephrotic syndrome. Most of the reports indicated that nephrotic syndrome developed at the same time as MCD, but in some cases MN was diagnosed several years after the onset of clinical signs suggestive of MCD [11, 14]. Two cases underwent multiple renal biopsies. In one, findings of MN had resolved after 2 years of treatment for MCD [7]. Granular deposition of IgG and C3, and to a lesser extent, IgM, was demonstrated in the first renal biopsy specimen of the case; however, immuno-staining was not performed in the second biopsy specimen [7]. The other was evaluated for disease activity after 3 years of refractory MN to strengthen the treatment strategy [14]. In the case, positive IgG, IgA, and C3 granular deposition in the first renal biopsy and the same staining pattern in the second biopsy was shown [14]. In our report, the patient underwent pathological evaluations of disease activity at an interval of 13 years. Granular deposits of IgG, IgA, and IgM in glomerular capillaries remained between the previous and the latest renal specimens, and the stage of MN had progressed. Immuno-histological findings in these cases implied that the reversible pathological changes that result in the removal of long deposited subepithelial and intermembranous immunoglobulins are difficult to detect in MN.

Generally, the etiology of idiopathic CD is unknown, but the recognized central factors include lymph node hyperplasia with polyclonal B lymphocyte expansion and cytokine storm (IL-6 and VEGF) [15]. The heterogeneity of the clinical course and renal involvement may be due to the pleiotropic actions of IL-6. A possible explanation is that overproduction of IL-6 may induce glomerulonephritis such as MN via polyclonal hypergammopathy and a variety of autoantibodies [10]. Although we could not directly confirm changes in the titer of blood PLA2R antibodies in this case, it was suggested that the serial increase in total IgG concentration contributed to the increased production of PLA2R antibodies and resulted in the recurrence of MN. Some CD cases respond well to corticosteroid monotherapy, and tocilizumab to counter excessive IL-6 production and other immunosuppressive agents can also effectively control disease activity when the effects of corticosteroid are incomplete. Renal involvement in CD cases often improves with treatment for CD [4]. The MCD consensus guidelines recommend anti-IL-6 monoclonal antibody as the first-line therapy for patients with severe CD, regardless of renal involvement [16]. However, the treatment strategy for MN in CD with a mild phenotype has not been established. An overview of past case reports showed that in one case with unicentric CD, MN improved only with resection of the affected lymph node [7]. Another case improved with corticosteroid monotherapy [4]. In most cases, improvement was achieved by tocilizumab [8, 10–14] or other immunosuppressive agents such as cyclophosphamide [4, 7, 9, 13], mizoribine [14], and cyclosporine [9, 14] in combination with corticosteroids. In our case, induction remission therapy with corticosteroids alone for 6 months proved effective, but it was insufficient for remission of both MN and CD, and the decision was made to add anti-IL-6 therapy in another facility.

Of special note in this case was the rarity of coincident MN and MCD, as the literature review of 11 cases also revealed, and no previous reports described results from renal biopsies taken prior to the development of MCD. As a limitation of this case report, we do not have any evidence to explain the causal links between previously diagnosed MN and newly developed CD, other than speculation that the long-term (38 years) PLA2R related primary MN was recurred by superimposition of plasma cell-driven inflammation and overproduction of antibodies. Another limitation of this study was the absence of examination for human herpesviurs-8 as a differential diagnosis for MCD.

To the best of our knowledge, this represents the first report of CD coincident with previously diagnosed MN. This case does not provide insights into the causal mechanisms of the pathophysiology, but may be worth considering possible involvement of CD pathology as a trigger for primary MN recurrence.

## Figures and Tables

**Table 1:** Laboratory findings on admission.

Urinalysis	
Specific gravity	1.019
Protein	4+
Blood	—
Sediment	
Red blood cells	1–4/HPF
White blood cells	<1/HPF
Urinary biochemical tests	
Daily urinary protein	6.2 g/24 h
Selectivity index	0.2
Bence-Jones protein	Negative
Complete blood count	
White blood cells	5700/mL
Neutrophils	45%
Eosinophils	12%
Basophils	1%
Lymphocytes	6%
Monocytes	36%
Hemoglobin	9.8 g/dL
Platelets	23.8 × 10^4^/mL
Blood biochemistry	
Total protein	6.6 g/dL
Albumin	1.4 g/dL
Uric acid	5.3 mg/dL
Urea nitrogen	10.4 mg/dL
Creatinine	0.83 mg/dL
Sodium	138 mmol/L
Chloride	107 mmol/L
Potassium	4.0 mmol/L
Corrected calcium	10.1 mg/dL
Phosphate	3.5 mg/dL
Blood biochemistry (cont.)	
Total bilirubin	0.2 mg/dL
Aspartate aminotransferase	27 U/L
Alanine aminotransferase	19 U/L
Lactate dehydrogenase	294 U/L
Alkaline phosphatase	170 U/L
Creatine kinase	54 U/L
Total cholesterol	125 mg/dL
LDL cholesterol	64 mg/dL
Triglycerides	102 mg/dL
Glucose	129 mg/dL
Hemoglobin A1c	5.9%
Serology	
C-reactive protein	0.63 mg/dL
HBs antigen	Negative
Anti-HCV antibody	Negative
Immunoglobulin G	2894 mg/dL
Immunoglobulin A	450 mg/dL
Immunoglobulin M	253 mg/dL
Immunoglobulin G4	672 mg/dL
Complement 3 (50–98)	75 mg/dL
Complement 4 (18–49)	7 mg/dL
CH50 (23–46)	33.0 U/mL
Antinuclear antibody	<×40
Cryoglobulin	(−)
Tumor markers, infection	
CEA (0.0–3.4)	3.2 ng/mL
CA19-9 (0–37)	44 U/mL
SCC (0.6–2.5)	3.5 ng/mL
Pro GRP (0–747)	50.7 pg/mL
sIL-2R (122–496)	2894 *U*/mL
T-SPOT	Negative

LDL, low-density lipoprotein; HBs, hepatitis B surface; HCV, hepatitis C virus; CH50, 50% hemolytic unit of complement; CEA, carcinoembryonic antigen; CA19-9, carbohydrate antigen 19-9; SCC, squamous cell carcinoma; GRP, gastrin releasing peptide; sIL-2R, soluble interleukin-2 receptor; T-SPOT, T cell spot test for tuberculosis; HPF, high-power field.

## Data Availability

The data generated during the current case are available from the corresponding author upon reasonable request.
